# Supplementary figures and images for: Automated high-throughput light-sheet fluorescence microscopy of larval zebrafish
Source: PLoS One. 2018 Nov 14;13(11):e0198705. doi: 10.1371/journal.pone.0198705 (PMC6235235; doi:10.1371/journal.pone.0198705)

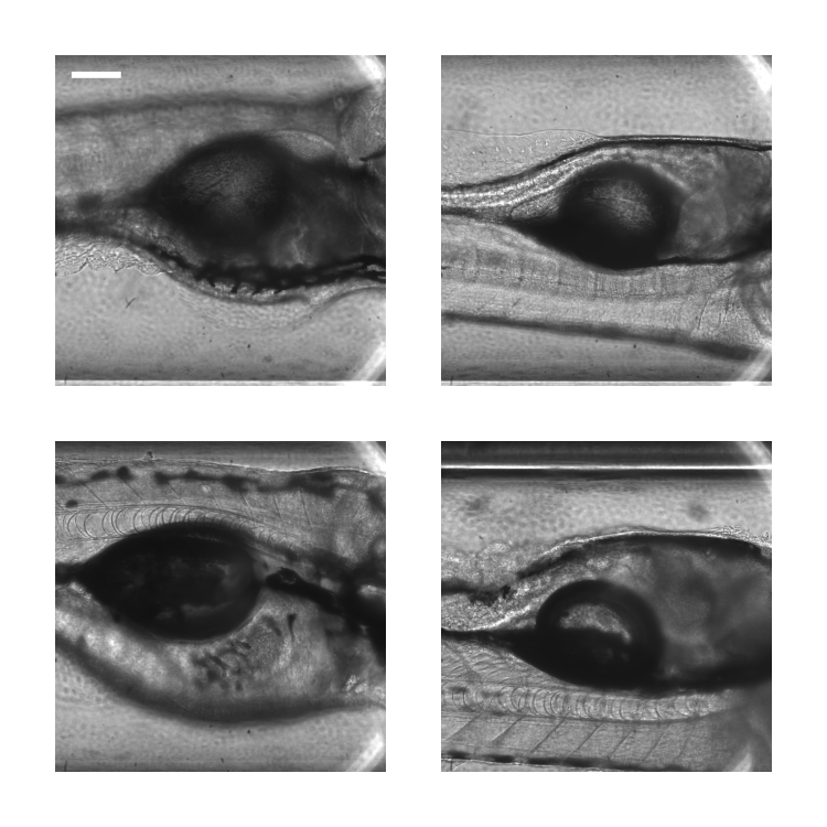

Supplement: S1 Fig — Sample brightfield images of four larval zebrafish, captured and saved prior to light sheet fluorescence imaging. Notably, the orientation is random in the capillary, with a tendancy toward either “gut down” or “gut up.” Scale bar: 100 μm. (TIF) [file pone.0198705.s001.tif]

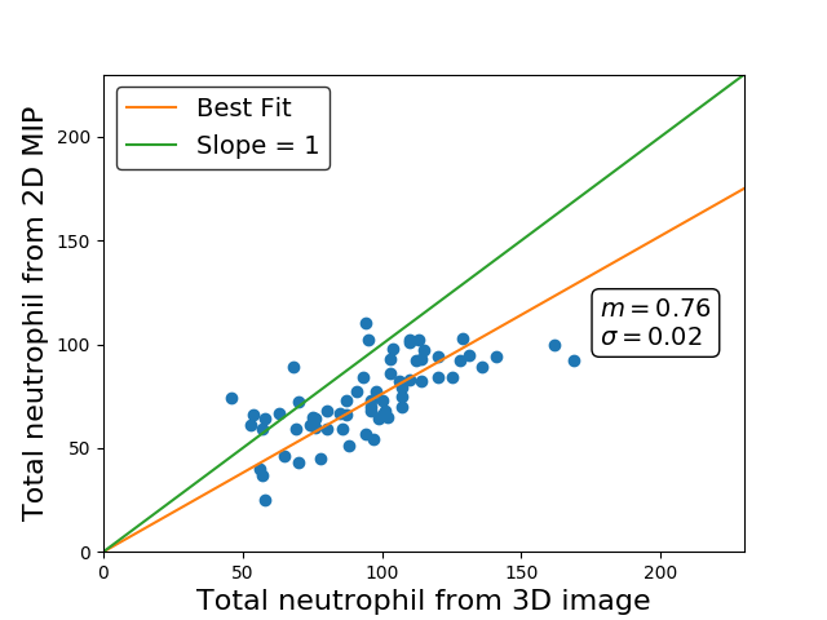

Supplement: S2 Fig — The number of neutrophils in 67 larval zebrafish, assessed from two-dimensional maximum intensity projections and from the full three-dimensional light sheet fluorescence scans. The former is 0.76 ± 0.02 of the latter, indicating that three-dimensional imaging is necessary to capture all the cells in a three-dimensional volume. (TIF) [file pone.0198705.s002.tif]
