# Supplementary material for: Automated high-throughput light-sheet fluorescence microscopy of larval zebrafish
Source: PLoS One. 2018 Nov 14;13(11):e0198705. doi: 10.1371/journal.pone.0198705 (PMC6235235; doi:10.1371/journal.pone.0198705)
Supplement: S1 Table — A parts list for the instrument, also indicating labels corresponding to Fig 1. (PDF) [file pone.0198705.s005.pdf]

S1 Table: Parts List

Automated High-Throughput Light-Sheet Fluorescence Microscopy of Larval Zebrafish

Savannah L. Logan, Christopher Dudley, Ryan P. Baker, Michael J. Taormina, Edouard A. Hay, Raghuveer Parthasarathy

Label in Fig 1	Part and Description	Supplier / Manufacturer	Part no.
**Excitation Optics**			
**1**	Lasers: 458nm, 488nm, 561nm, 594nm; 20 mW	Coherent	Saphire
	Fiber launch	Thorlabs base + custom objective mount + Siskiyou fiber mount	
	Objective lens for fiber launch	Zeiss	5x plano-apochromat
**2**	Acousto-optic tunable filter (AOTF)	AA Opto-electronic	AOTFnC-VIS-TN 1001 / 340697
	Fiber switch (fiber = Nufern S460 HP)	Leoni	eol 1x4
**3**	Cage Mount for galvanometer mirror, 30mm	Thorlabs	GCM001
**3**	Galvanometer mirror	Cambridge Technology	6210H
**4**	PrePrism-sample chamber objective	Mitutoyo Plan APO 5X	Z03154214
**Hardware related to the sample chamber**			
	Sample chamber: PA11 Black plastic, 3D printed	Protolabs	3D printed
	Alternate sample chamber (blue): E-Shell 3000 biocompatible plastic	Envisiontec.com	3D printed
	Objective lens: Olympus 20x/0.5 Water Immersion Obj.	Edmund Optics	34556
	O-rings,various	McMaster-Carr	various
	Glass window, 20mm diameter, 2mm thick, VIS-NIR fused silica	Edmund Optics	49-642
	Heater: flex heater, sheet of ~10	Omega.com	KH-kit-efh-15001
**5**	Prism, 20mm, N-BK-7 rt angle prism	Edmund Optics	32-334
**5**	Prism mount: mini-translation stage, one axis, linear	ThorLabs	DT12
**5**	Prism holder	UO	custom
**5**	45° mount	Edmund Optics	59001
**5**	1.5" straight mirror mount	Edmund Optics	33497
**5**	1.0" straight mirror mount, mount only	Edmund Optics	33-501
	English long travel rack & pinion, camera adjustment	Edmund Optics	59-333
	One knob stage	Edmund Optics	59-331
	Rail, 66mm square	ThorLabs	XT66
	Rail, Dovetail	ThorLabs	XT66SP
	Rail carriages	ThorLabs	XT66P2
	6mm rods for cage system/sample chamber holder	ThorLabs	ER-xx, various lengths
**Fluidics**			
	Flow cell and holder		
**11c**	Flow cell: 3/16" dia. Clear acrylic rod	McMaster-Carr	UO Machine shop
**11b**	Flow cell: 1/2" blunt stainless steel needle 18gauge	McMaster-Carr	75165A675
**11d**	Flow cell: Round glass capillary, 50mm, 0.7mm ID, 0.87mm ID	Vitrocom Technical Glass	cv7087-B
	Flow cell: Square glass capillary, 50mm, 0.7mm ID, 0.140mm Wall	Vitrocom Technical Glass	8270-050
	Flow cell: Tub and Tile Silicon caulk	Hardware store	
**11a**	Tubing, "Silastic," 0.74mm ID, Platinum cured	Fisher Scientific	11-189-15C
	Luer lock plastic valve		
**9**	Syringe Pump, (BenchTop Style) 1/10 ml glass syringe	KD scientific	111
	Metal Y tube, 0.05" 0.033" 1/4"	ztubes.com	HSCY-18
	Relay (for valves), 60 VDC/3.5A, 5Volt Logic	Grayhill	70G-0DC5x16
**10**	12V solenoid pinch valves (x6) 15PSI PN98302	Cole Parmer	
**10**	N.O. Pinch Valve, 0.75" dia., 0.030" ID-0.065" OD Tubing, 12 VDC	Clippard	N PV1-1O-01-122
**10**	N.C. Pinch Valve, 0.75" dia., 0.030" ID-0.065" OD Tubing, 12 VDC	Clippard	NPV1-1C-01-122
**10**	N.C. Pinch Valve, 15psi 12V	ColePalmer	98302-02
**Other Hardware**			
**8**	Hamamatsu Orca Flash 4.0 Camera	Hamamatsu	C11440
	Firebird CL 2011 camera link board	Active Silicon	AS-FBD-1XCLD-2PE8
**6**	LED light (Bright field illumination)	ThorLabs	MCWHL5
	LED driver	ThorLabs	LEDD1B, M00400850
	Stage controller	Applied Scientific Instrumentation	TG-1000-16, TG16-XY:5A-Z:5U
	xyz stage	Applied Scientific Instrumentation	LS-5507,+?
	xyz arm, 8020, 1" x ~12" long aluminum channel	McMaster-Carr	47065T101
	Data Acquisition (DAQ) Board	National Instruments	USB-6343 X-eries
